# Spectral Relationships of ZnPc and CuPc: UV-VIS and Fluorescence Behavior in Liquids and Thin Films

**DOI:** 10.3390/molecules30214262

**Published:** 2025-10-31

**Authors:** Vadim Morari, Ion Lungu, Victor Suman, Lidia Ghimpu, Tamara Potlog, Radu Tigoianu, Iuliana Stoica, Carmen Gherasim, Anton Airinei

**Affiliations:** 1 D. Ghitu Institute of Electronic Engineering and Nanotechnologies, Technical University of Moldova, Academiei Str. 3/3, 2028 Chisinau, Moldova; vadim.morari@iien.utm.md (V.M.); victor.suman@iien.utm.md (V.S.); lidia.ghimpu@iien.utm.md (L.G.); 2 Laboratory of Organic/Inorganic Materials for Optoelectronics, Moldova State University, 60 Al. Mateevici St., 2009 Chisinau, Moldova; ionlungu.usm@gmail.com (I.L.); tpotlog@gmail.com (T.P.); 3 Laboratory of Physical Chemistry of Polymers, “Petru Poni” Institute of Macromolecular Chemistry, 41A Grigore Ghica Voda Alley, RO-700487 Iasi, Romania; tigoianu.radu@icmpp.ro (R.T.); stoica_iuliana@icmpp.ro (I.S.); gherasim.carmen@icmpp.ro (C.G.)

**Keywords:** ZnPc, CuPc, UV-VIS absorbance, reflectance, fluorescence emission, AFM

## Abstract

In this study, zinc phthalocyanine (ZnPc) and copper phthalocyanine (CuPc) thin films fabricated by drop casting (DC) and close space sublimation (CSS) have been investigated and compared with ZnPc and CuPc solutions in formic acid (29 µmol/L). The results show that the CSS method produces films with improved molecular ordering, enhanced surface uniformity, superior optical and morphological properties compared to those obtained by drop casting. Moreover, CSS allows a precise and reproducible deposition, resulting in thinner, homogeneous layers with strong substrate adhesion and fewer defects. Optical characterization confirms that CSS films display high transparency (~90%), a sharp Q-band around 680 nm, and a fluorescence maximum at ~825 nm with the strongest emission intensity.In contrast, DC films show lower transparency (<70%), a slightly shifted Q-band (~675 nm), and similar emission around 825 nm. The fluorescence is strongly thickness-dependent: at ~100 nm, the emission band appears at 795 nm, while films thicker than 300 nm exhibit a red-shifted maximum at ~825 nm. AFM analysis further demonstrates the influence of deposition method: CSS yields smoother films with tunable morphology, while DC produces rougher, less controllable surfaces. Overall, CSS is shown to be the more effective approach for fabricating high-quality phthalocyanine films for optoelectronic applications such as photovoltaics and sensors.

## 1. Introduction

In a world where technology advances rapidly, materials forming the foundation of organic electronic devices are becoming increasingly important. Among these, two remarkable chemical compounds, ZnPc (zinc phthalocyanine) and CuPc (copper phthalocyanine), play essential roles in developing innovative technologies, from organic solar cells [1,2], transistors to LEDs [3] and sensors [4,5,6]. Although both belong to the same family of organic compounds, subtle differences between these two metal-centered molecules can significantly impact their performance in various applications.

**Figure 1:**
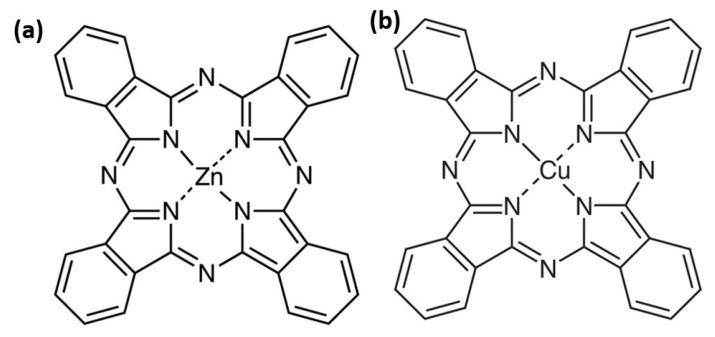
Chemical structure of the ZnPc (**a**) and CuPc (**b**).

These metal phthalocyanines share a similar molecular structure, but differ by the central metal atom: zinc for ZnPc and copper for CuPc (Figure 1). These small changes deeply affect the molecule’s electronic density, geometry, electron transport properties, as well as its optical, electrical, and chemical behavior. Even in seemingly simple environments such as thin films or liquids, typical of cutting-edge technologies, each molecule reveals unique features essential for device performance. Metal phthalocyanines are planar macrocyclic organic compounds characterized by an aromatic ring coordinating a central metal atom [7].

While ZnPc is renowned for its thermal stability and ability to absorb visible light efficiently, CuPc stands out for its versatility in photovoltaic applications and excellent environmental stability.

ZnPc typically adopts a rigid planar geometry with a Zn^2+^ ion that imparts high molecular stability and rigidity. In contrast, CuPc, containing a Cu^2+^ ion, may exhibit slight distortions from planarity due to spin interactions and copper’s electronic configuration. Table 1 shows the most important physical properties of ZnPc as compared to CuPc.

The UV-Vis spectra of ZnPc and CuPc exhibit two major absorption bands: the Q-band (in the 600–700 nm range) and the Soret band (300–400 nm range) [19]. The Q-band is crucial for photovoltaic applications, as it corresponds to the absorption in the visible spectrum. ZnPc features a strong, relatively narrow Q-band centered around 675 nm [20]. Its fluorescence quantum yield can reach up to 10%, making it highly suitable for optical sensors and luminescent devices. It is favored in organic solar cells and organic LEDs due to its efficient light absorption and fluorescence. Its thermal stability enables compatibility with higher-temperature fabrication processes. CuPc displays a slightly blue-shifted and broader Q-band around 630 nm, with notably weak fluorescence due to efficient non-radiative decay pathways, which contributes to its enhanced photostability [21,22]. It is commonly used in organic transistors, solar cells, and environments demanding long-term durability. Although, its photovoltaic efficiency may be slightly lower than ZnPc, its robustness in harsh conditions makes it invaluable. Key challenges for both materials include optimizing electrode interfaces, controlling film thickness and morphology precisely, and integrating them into multilayer device architectures without performance loss. A comprehensive review by Gould provides a detailed overview of the structural and electrical conduction properties of the evaporated phthalocyanine thin films. It emphasizes the influence of crystalline phases (α and β), film thickness, and substrate temperature on film structure, as well as the impact of contact type and surface states on charge transport. The authors conclude that further advancements in this field rely on improved material purity, refined deposition techniques, and enhanced structural/electrical characterization, given the sensitivity of phthalocyanine films to environmental factors such as oxygen absorption [23]. This article explores the differences and similarities between ZnPc and CuPc, aiming to better understand how their optical absorption and fluorescence spectra can influence properties in thin films. Many methods have been employed to fabricate thin films of zinc phthalocyanine (ZnPc) and copper phthalocyanine (CuPc), including thermal evaporation [24], pulsed laser deposition (PLD) [25], Langmuir–Blodgett deposition [26], physical vapor deposition (PVD) [27], atmospheric pressure chemical vapor deposition (APCVD) technique [28], electrodeposition method [29]. However, these techniques are often resource-intensive, requiring complex instrumentation and high operational costs. In this study, we focus on two simpler and more cost-effective solution-based approaches: Close-spaced sublimation (CSS) and drop casting (DC) methods. We aim to investigate the optical properties of the resulting thin films, along with their surface morphology and roughness, characterized using Atomic Force Microscopy (AFM). By analyzing these characteristics, we aim to provide insights into how material composition affects films properties. This study seeks to contribute to the optimization of optoelectronic applications through a detailed comparison of these compounds. Additionally, understanding the interplay between molecular structure and film morphology could lead to improved fabrication techniques. The findings may also guide future research in tailoring material properties for specific technological uses. Particular attention will be given to the surface roughness of films deposited via the CSS method, as roughness plays a critical role in light scattering and charge transport at the interfaces. The CSS technique, known for producing high-quality, dense films with controlled thickness, offers advantages in scalability and uniformity, making it a promising approach for fabricating organic semiconductor layers.

## 2. Results and Discussion

### 2.1. UV-VIS and Fluorescence Spectra of ZnPc and CuPc Solution

**Figure 2:**
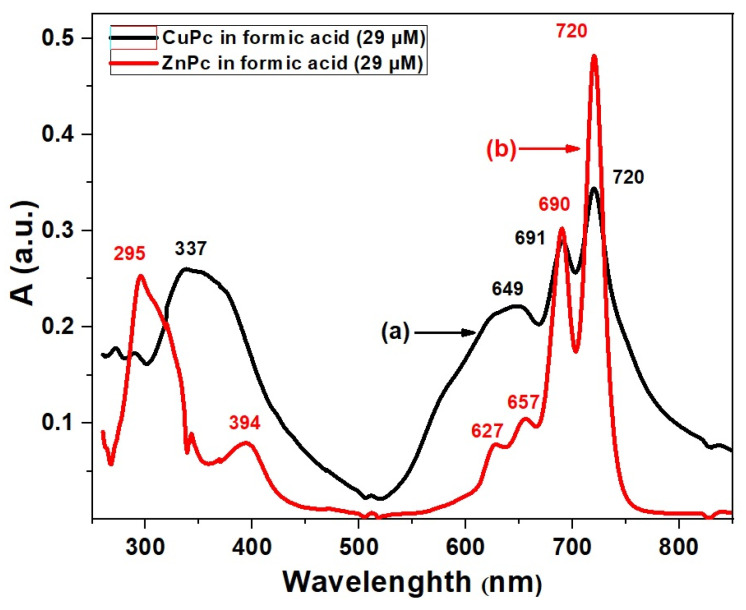
UV-VIS spectra of CuPc (a) and ZnPc (b) solutions synthesized in formic acid (FA).

In this work, we have restricted our study to a fixed concentration (29 μM in formic acid) in order to allow a direct comparison between ZnPc and CuPc. Figure 2 shows the UV-VIS absorption spectra of CuPc and ZnPc in formic acid at this concentration, where both compounds exhibit the characteristic Soret and Q absorption bands, whose detailed differences provide insights into their electronic structures and potential applications [30].

For ZnPc, the Soret band shows two distinct maxima at 295 nm and 394 nm, indicating strong high-energy π–π* transitions associated with the phthalocyanine ring influenced by the zinc ion [31]. The Q band region reveals four absorption bands at 627 nm, 657 nm, 690 nm, and 720 nm. This multiplicity in Q bands suggests notable vibronic coupling and possible molecular aggregation or specific solvent interactions [32]. The complex Q band pattern can enhance light-harvesting efficiency by broadening the absorption range, which is beneficial for photovoltaic and sensor applications [33], where a broad spectral coverage is desired. In contrast, CuPc presents a single Soret band maximum at 337 nm, red-shifted relative to ZnPc, reflecting a distinct electronic environment caused by the copper center’s d-orbital configuration [34]. Its Q bands occur at 649 nm, 691 nm, and 720 nm, fewer than those of ZnPc. The simpler Q band structure may indicate less vibronic splitting or reduced aggregation in solution, which can lead to more defined and stable optical properties. This behavior is advantageous for applications requiring sharp and stable absorption features, such as precise optical sensors or photonic devices [35]. The red-shift in both Soret and Q bands in CuPc compared to ZnPc suggests that copper’s electronic influence stabilizes the excited states differently, modifying absorption energies. The differences in vibronic structure and aggregation behavior between ZnPc and CuPc reflect their distinct coordination chemistry and interaction with formic acid solvent, which can be exploited to tailor material properties for specific optoelectronic applications [36].

**Figure 3:**
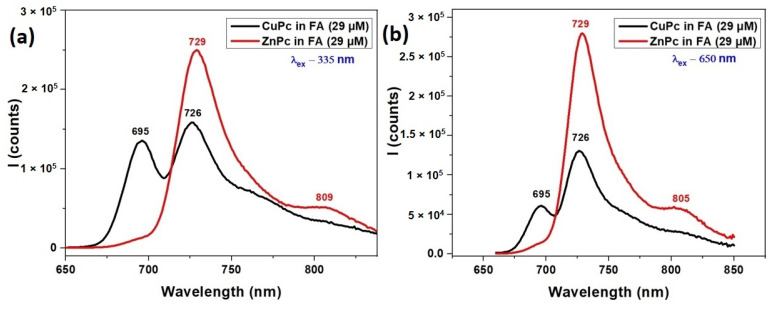
Fluorescence spectra of CuPc and ZnPc solutions synthetized in formic acid (FA) λ_ex_ = 335 nm (**a**) and λ_ex_ = 650 nm (**b**).

Figure 3 illustrates the fluorescence spectra of CuPc and ZnPc dissolved in formic acid (FA), under two different excitation wavelengths 335 nm and 650 nm. These spectra provide complementary insights to the UV-VIS absorption features shown in Figure 2, offering a more complete understanding of the photophysical behavior of these metallophthalocyanines in solution.

Under excitation at 335 nm (Figure 3a), which corresponds to the Soret band region, both ZnPc and CuPc exhibit emission in the near-infrared region. ZnPc shows a strong fluorescence peak at 729 nm and a weaker shoulder at 809 nm, indicating efficient relaxation from higher excited states to the emissive Q band region. This emission profile aligns well with its Q-band absorption (627–720 nm), suggesting effective energy transfer and minimal non-radiative losses.

CuPc, under the same excitation, displays fluorescence peaks at 695 nm and 729 nm. The emission at 695 nm corresponds closely with its Q band absorption maximum at 691 nm (Figure 2), while the 729 nm peak may arise from vibronic transitions or aggregation-related emissive states. The fluorescence from CuPc is generally weaker and slightly more blue-shifted compared to ZnPc, consistent with CuPc’s typically lower fluorescence quantum yield due to enhanced intersystem crossing induced by the paramagnetic copper ion.

At an excitation wavelength of 650 nm (Figure 3b), which directly excites the Q band region, ZnPc again shows strong emission at 729 nm with an additional shoulder at 805 nm, reinforcing its efficient radiative relaxation from the Q band excited states. The 805 nm peak suggests either vibrational relaxation or minor aggregation-related effects influenced by the FA solvent.

CuPc, excited at 650 nm, shows fluorescence at 695 nm and 726 nm, closely mirroring the Q band absorption features noted in Figure 2. However, the emission intensity is generally weaker than ZnPc, and the narrower range again points to CuPc’s more limited emissive capacity, likely due to non-radiative decay pathways prevalent in Cu-based phthalocyanines.

The fluorescence spectra confirm and extend the absorption findings from Figure 2. ZnPc demonstrates stronger and broader emission under both excitation conditions, indicative of higher fluorescence efficiency and more complex relaxation dynamics. CuPc emits weakly, with peaks aligned closely to its absorption maxima, consistent with its lower quantum yield. The interplay between absorption and fluorescence behaviors highlights the key roles of the central metal ion, excitation wavelength, and solvent environment in tuning the optical responses of metallophthalocyanines for optoelectronic and sensing applications.

**Figure 4:**
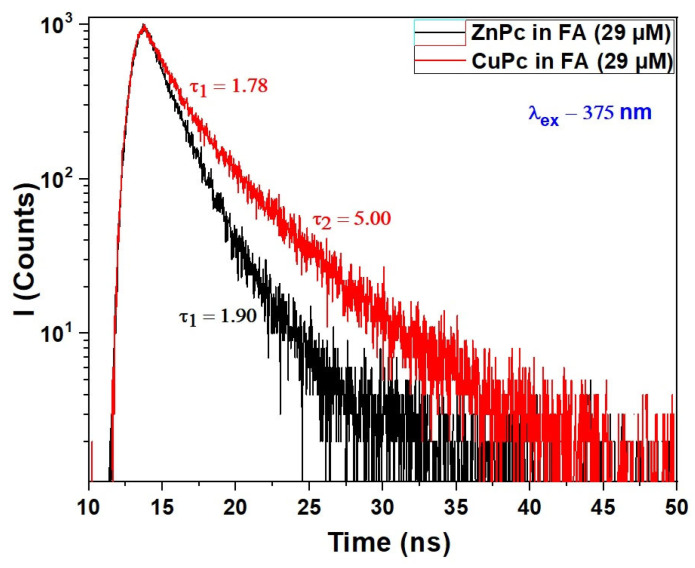
Fluorescence decays of CuPc and ZnPc solutions synthesized in formic acid (FA) at wavelength excitation of λ_ex_ = 375 nm.

Figure 4 illustrates the fluorescence decay profiles of Copper Phthalocyanine (CuPc) and Zinc Phthalocyanine (ZnPc) dissolved in formic acid (FA), following excitation at 375 nm.

The decay dynamics provide crucial insight into the excited-state behavior of these molecules, complementing the UV-VIS absorption spectra discussed earlier. For ZnPc, a single exponential decay is observed with a fluorescence lifetime of 1.90 ns, indicating a relatively uniform and well-defined emissive state in FA. This suggests that ZnPc remains largely in a monomeric or weakly interacting state under these conditions, consistent with the distinct vibronic features observed in its Q-band absorption. The well-resolved fluorescence decay implies minimal aggregation or quenching effects in this solvent environment.

In contrast, the fluorescence decay of CuPc exhibits a biexponential behavior, with two distinct lifetimes: 1.78 ns and 5.00 ns. The presence of two decay components indicates that CuPc exists in at least two emissive states or environments when dissolved in formic acid. The shorter lifetime (1.78 ns) likely corresponds to a monomer-like species or a weakly interacting form, similar in behavior to ZnPc. Meanwhile, the longer component (5.00 ns) may result from aggregated species, excimers, or solvent-stabilized complexes that prolong the excited-state lifetime. This duality in fluorescence response aligns with the more limited and slightly red-shifted Q-band absorption spectrum of CuPc, suggesting a different aggregation or solvation dynamic compared to ZnPc.

Formic acid again plays a key role by influencing solvation and potential aggregation states. Its polarity and capacity for hydrogen bonding may stabilize different molecular conformations or facilitate weak dimerization, especially in CuPc, leading to complex excited-state behavior. The sensitivity of CuPc’s fluorescence lifetime to its environment further supports the hypothesis that the central metal ion affects not just ground-state absorption but also relaxation dynamics in the excited state.

These photophysical differences are highly relevant for optoelectronic applications. ZnPc, with a single, fast-decaying fluorescence component, is advantageous in systems where predictable, rapid deactivation is desired, such as time-resolved sensing or fast switching. CuPc, on the other hand, may be more suitable in applications where long-lived excited states are beneficial, such as in triplet harvesting or energy transfer systems.

### 2.2. Comparison of UV-VIS and Fluorescence Spectra of ZnPc and CuPc Thin Films Deposited by Different Methods

If we compare the absorption and fluorescence spectra in formic acid solution with those obtained in films, several important differences emerge due to the change in the physical environment and molecular interactions.

Below, we present the UV-VIS absorption spectra of thin films of copper phthalocyanine (CuPc) and zinc phthalocyanine (ZnPc), fabricated using two deposition techniques: drop casting and CSS method, at two distinct substrate temperatures. The spectral features reveal significant differences in molecular organization and optical behavior based on the deposition technique. In general, ZnPc and CuPc films exhibit broader and red-shifted Q-bands compared to their solution spectra, indicating enhanced molecular aggregation in the solid state. However, the degree and nature of this aggregation depend strongly on the fabrication method.

**Figure 5:**
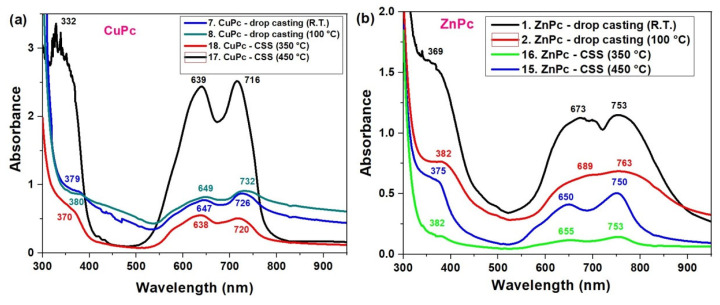
UV-VIS spectra of CuPc (**a**) and ZnPc (**b**) films obtained by CSS and DC methods.

In the CuPc films (Figure 5a), the spectra reveal a strong dependence on both the deposition method and temperature. The film deposited via CSS at 450 °C (black curve) exhibits two well-resolved Q-band peaks at 639 nm and 716 nm, indicating a high degree of molecular ordering.

This spectral profile is characteristic of H-type aggregation, where strong π–π stacking leads to split absorption features. In contrast, the drop-casted films, especially at room temperature (blue curve), show broader and less intense bands, with Q-band maxima shifted to 647 nm and 726 nm. This suggests a more disordered molecular arrangement and increased aggregation variability. As the drop casting temperature increases to 100 °C (green curve), the Q-band becomes slightly more resolved (649/732 nm), showing partial improvement in ordering. At lower CSS temperature (350 °C, red curve), CuPc shows peaks at 638 nm and 720 nm, indicating a compromise between order and aggregation, but still more structured than drop casting. The UV region also shows shifts: for instance, the Soret band (B-band) shifts from 379 nm in drop-casted films to 366 nm in the CSS deposited films at 350 °C, reflecting differences in electronic transitions due to molecular packing.

In the ZnPc films (Figure 5b), a similar trend is observed. The drop-casted film at room temperature (black curve) has strong, broad Q-bands centered at 673 nm and 753 nm, and a pronounced B-band at 369 nm, which are typical of J-aggregate behavior and significant intermolecular interaction. The higher UV absorbance observed for ZnPc films prepared by drop casting at room temperature is mainly due to their larger thickness and inhomogeneous morphology, as the slower evaporation of the solvent allows for the deposition of a larger amount of material, which was not observed for CuPc films. Heat treatment at 100 °C leads to thinner and more uniform films with better molecular ordering, which can reduce the apparent absorbance. Therefore, the lower absorbance of heat-treated films does not imply a poorer material quality or performance. However, the spectrum becomes more structured and blue-shifted with improved processing. CSS-deposited films, especially at 450 °C (blue curve), show clearer peaks at 650 nm and 750 nm, while the 350 °C CSS sample (green curve) reveals Q-band peaks at 655 nm and 753 nm.

Notably, the shift and sharpening of peaks in the CSS films, particularly the reduction in peak overlap and the emergence of distinct vibronic features and better π–π stacking [37], can occur.

Overall, these data confirm that CSS at elevated temperatures enhances the optical quality of CuPc and ZnPc films by promoting molecular ordering, reducing random aggregation, and generating sharper, more defined Q-band features. These changes directly affect the films’ suitability for optoelectronic devices, such as organic photovoltaics, where controlled light absorption and minimal fluorescence quenching are critical.

**Figure 6:**
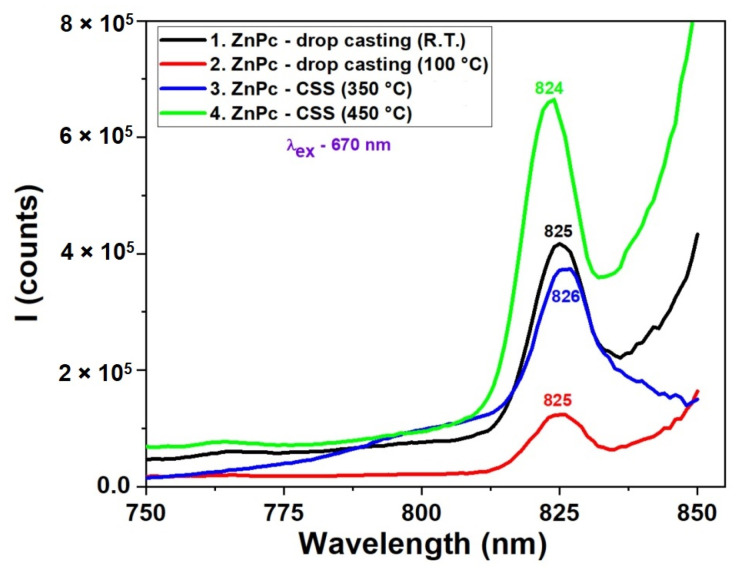
Fluorescence spectra of ZnPc thin films obtained by DC at room temperature and 100 °C and by CSS at 350 °C and 450 °C.

The fluorescence spectra presented in Figure 6 reveal the strong influence of deposition technique, temperature, and time on the photophysical behavior of ZnPc thin films. Films prepared by the CSS method at 450 °C for 3 min exhibit the most intense and sharp emission peak around 824 nm, suggesting enhanced molecular ordering, which favors radiative recombination. In contrast, films obtained via drop casting or CSS with shorter deposition times (1 min) show significantly lower fluorescence intensity and broader peaks, indicating the presence of non-radiative recombination centers, likely due to increased disorder or incomplete film formation. The spectral position of the secondary emission peak remains relatively consistent (~824–826 nm) for most samples, confirming that the molecular structure of ZnPc remains intact, while variations in intensity reflect changes in film quality and morphology. These results demonstrate that optimized thermal conditions and deposition time in CSS significantly enhance the optoelectronic performance of ZnPc films, making them more suitable for photonic and photovoltaic applications. When comparing the fluorescence spectra in Figure 6 with the UV-VIS absorption spectra (Figure 5), a correlation can be observed between film quality, optical absorption, and emission behavior. In the absorption spectra, ZnPc films deposited by CSS at higher temperatures, particularly at 450 °C, exhibited more intense and sharper Q-band absorption features, indicative of stronger π–π stacking and improved molecular ordering. This structural organization directly enhances the radiative recombination efficiency, as evidenced by the higher fluorescence intensity at ~824 nm in the corresponding emission spectrum.

Conversely, films prepared by drop casting or with shorter CSS durations showed broader, less defined absorption bands and correspondingly weaker fluorescence. This suggests that the absorption strength and sharpness, especially in the Q-band region, can be predictive of the fluorescence performance. The alignment of the absorption and emission trends supports the conclusion that improved molecular ordering enhances light-harvesting efficiency and minimizes non-radiative decay pathways, making such films more promising for optoelectronic applications.

**Figure 7:**
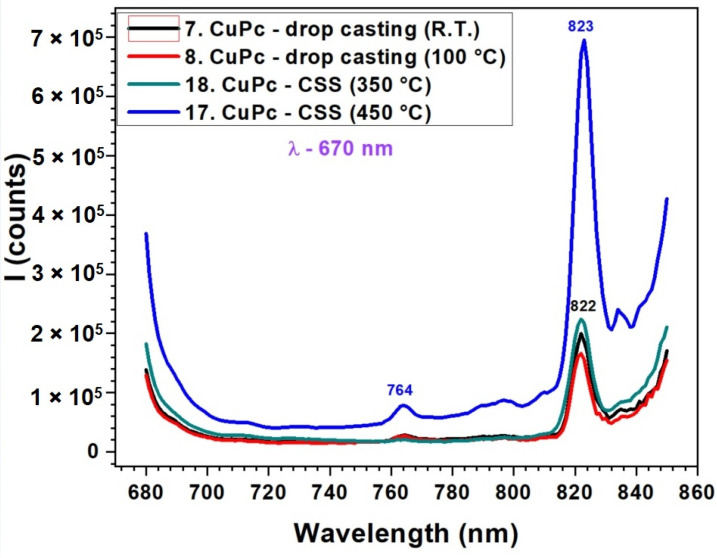
Fluorescence spectra of CuPc thin films obtained by DC at room temperature and 100 °C and by CSS at 350 °C and 450 °C.

The fluorescence spectra in Figure 7 correlated with the UV-VIS absorption data from Figure 5, reveal a strong relationship between the molecular organization inferred from absorption and the emission efficiency of CuPc films. In the absorption spectra, CuPc films prepared by CSS at 450 °C displayed more intense and narrower Q-band features, indicative of improved π–π stacking and reduced disorder. This enhanced molecular alignment facilitates more efficient radiative recombination, which is clearly reflected in the fluorescence spectrum as a strong, sharp peak at 823 nm. The additional peak observed at around 825 nm is indeed not attributed to the main fluorescence band but rather to secondary emission processes. In ZnPc and CuPc thin films, such red-shifted emission commonly arises from molecular aggregation effects, particularly the formation of J-aggregates or excimeric states due to π–π stacking interactions between adjacent molecules. These effects are more pronounced in thicker or highly ordered films obtained by the CSS method. Therefore, we ascribe the 825 nm feature to emission from aggregated species rather than to intrinsic fluorescence from the molecular singlet state. Conversely, drop-cast films and CSS films deposited at lower temperatures (350 °C) exhibited broader and less intense absorption bands. This suggests a less ordered molecular structure, which correlates with the significantly reduced fluorescence intensity and broader emission profiles in these samples. The small peak at 764 nm in the high-temperature CSS film may correspond to a vibronic transition or to emission from aggregated species, which become prominent only in well-ordered films. Below is presented a quantitative comparison table (Table 2) summarizing the key absorption (from Figure 5) and fluorescence (from Figure 6 and Figure 7) peak parameters for ZnPc and CuPc thin films under different deposition conditions.

To confirm the validity of the optical data, the absorption and fluorescence spectra of the ZnPc and CuPc thin films were compared with previously reported results for metal phthalocyanines. The ZnPc films display two well-defined Q-band absorption maxima at 673 and 753 nm, which are in excellent agreement with the characteristic Q-band positions reported for Zn (II)–phthalocyanine systems in both solution and solid states, typically ranging between 670 and 760 nm [38,39]. The CuPc films exhibit two distinct absorption maxima at 639 and 716 nm, consistent with the Davydov splitting observed in crystalline CuPc and indicative of α/β-phase coexistence, as previously described [40,41].

The slight spectral shifts and broadening detected in our samples compared to the literature are mainly attributed to molecular aggregation (J- or H-type), film thickness, and the influence of the deposition technique—drop casting and close space sublimation—on molecular packing and orientation at the substrate interface. In particular, films prepared by drop casting tend to favor random aggregation and broader absorption features, while those obtained by close space sublimation exhibit a higher degree of ordering and narrower bands, consistent with excitonic coupling and phase stabilization effects [42].

Regarding fluorescence, the ZnPc films present a pronounced emission band centered at 825 nm, while CuPc shows a weak fluorescence peak around 823 nm. These emission characteristics are consistent with the optical behavior typically reported for phthalocyanine thin films, where ZnPc retains a red/near-infrared emission and CuPc undergoes strong fluorescence quenching due to efficient intersystem crossing and enhanced nonradiative decay channels [43,44]. The good agreement of our data with the literature thus confirms the reliability of the experimental results and supports the interpretation of the observed spectral features in terms of molecular aggregation, film morphology, and deposition-dependent ordering.

**Figure 8:**
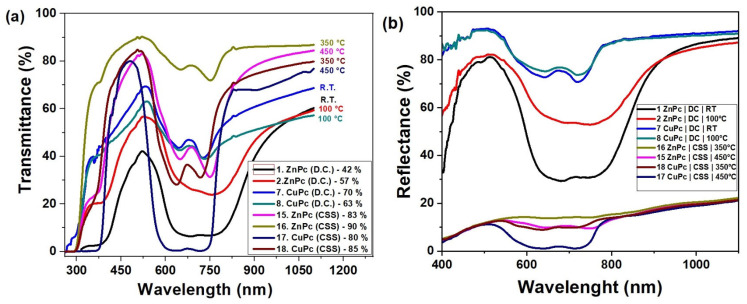
Transmittance (**a**) and reflectance (**b**) spectra of CuPc and ZnPc thin films.

Figure 8a illustrates the optical transmittance behavior of zinc phthalocyanine (ZnPc) and copper phthalocyanine (CuPc) thin films deposited by two different techniques: drop casting and close-spaced sublimation (CSS), over the spectral range of 300 to 1200 nm.

From the spectral curves, a clear distinction is observed between the two fabrication methods. Films prepared by drop casting (curves 1, 2, 7, and 8) exhibit significantly lower transmittance, with ZnPc films reaching values between 42% and 57%, and CuPc films between 63% and 70%. These lower transmittance levels are indicative of thicker, less uniform films, possibly with higher surface roughness or denser molecular packing that reduces optical transparency.

In contrast, films obtained via CSS (curves 15–18) show notably higher transmittance values, ranging from 80% to 90%. ZnPc films deposited at elevated temperatures (350–450 °C) reach up to 90%, suggesting improved structural uniformity and surface homogeneity. Similarly, CuPc films deposited under CSS conditions display transmittance up to 85%, reflecting superior optical quality compared to their drop-cast counterpart.

The observed increase in transmittance with temperature in CSS samples further supports the conclusion that substrate heating promotes better molecular ordering and film densification. The higher transparency of CSS films makes them suitable for optoelectronic applications where minimal light absorption is desired in specific layers, such as in photovoltaic devices, photodetectors, or optical coatings.

Overall, the data confirm that the deposition technique and thermal treatment play a critical role in tuning the optical properties of phthalocyanine-based thin films.

The reflectance spectra presented in Figure 8b reveal significant differences between the films deposited by DC and those obtained by CSS at elevated temperatures. Films prepared at RT and 100 °C by DC exhibit high reflectance values, reaching 70–80% in the visible range, suggesting a strong interaction of the incident radiation with the surface. In contrast, the films deposited by CSS at 350–450 °C are characterized by low reflectance, below 20%, indicating a more ordered structure and reduced roughness. This behavior is further supported by the transmittance spectra in Figure 8a, where the same CSS-grown films show high transmittance values, in the range of 80–90%, confirming their superior transparency. The DC-deposited films at low temperatures exhibit modest transmittance of 40–60%, which explains their increased reflectance and the associated optical losses.

A complementary picture is provided by the absorbance spectra in Figure 5, where characteristic Q- and B-bands of CuPc and ZnPc are clearly observed in the ranges 600–750 nm and 350–400 nm, respectively. These absorbance features correspond precisely to the minima observed in both transmittance and reflectance, confirming the strong light–matter interaction of phthalocyanines in these regions. For CuPc, distinct maxima appear around 639–716 nm, while ZnPc shows pronounced peaks near 673–753 nm, consistent with the lower transmittance and reflectance values in the same intervals. Films deposited by CSS exhibit sharper and more intense absorption bands compared to those prepared by DC, indicating improved molecular ordering and enhanced optical activity. In the Near-Infrared region (>800 nm), absorbance decreases while transmittance increases, with reflectance maintaining moderate values around 10–20%.

This overall complementarity between reflectance, transmittance, and absorbance spectra demonstrates that reducing reflectance is directly correlated with higher transmittance and enhanced absorption efficiency. Comparatively, the deposition methods and thermal treatments play a decisive role in balancing these optical properties. Therefore, CSS films obtained at elevated temperatures can be considered the most promising for optoelectronic applications due to their advantageous combination of high transparency, strong absorbance in the Q- and B-band regions, and low reflectance.

### 2.3. AFM Investigations

**Figure 9:**
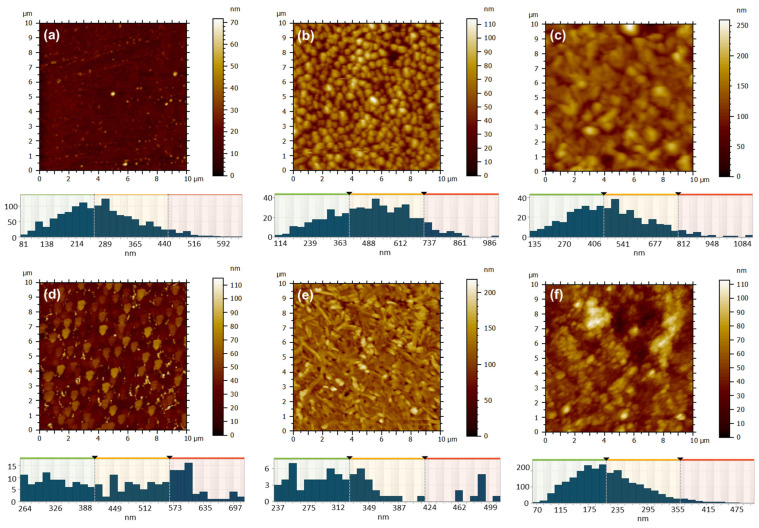
AFM 2D topographic images and corresponding grain-size distribution obtained by CSS method for the samples: 16 ZnPc-350 °C, t = 1 min (**a**); 15 ZnPc-450 °C, t = 1 min (**b**); 3 ZnPc-350 °C, t = 3 min (**c**); 4 ZnPc-450 °C, t = 3 min (**d**); 18 CuPc-350 °C, t = 1 min (**e**); 17 CuPc-450 °C, t = 1 min (**f**).

**Figure 10:**
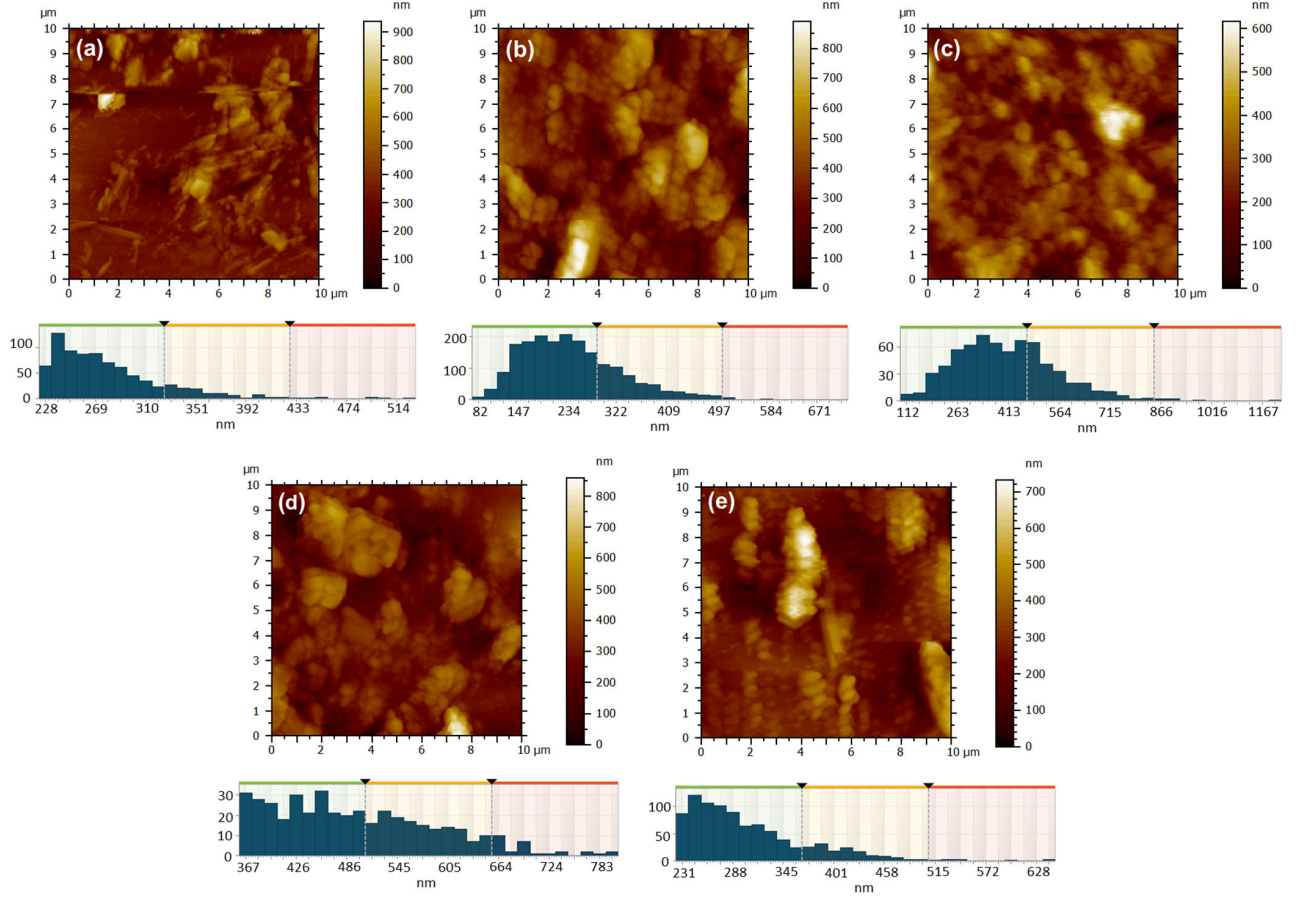
AFM 2D topographic images and corresponding grain-size distribution for the samples obtained by DC method: 1 ZnPc, RT (**a**); 2 ZnPc-100 °C (**b**); 13 ZnPc-350 °C (**c**); 7 CuPc, RT (**d**); 8 CuPc-100 °C (**e**).

Atomic force microscopy was employed to investigate the changes in surface morphology based on various film deposition techniques: close space sublimation (Figure 9) or drop casting (Figure 10), along with factors such as evaporator temperature (350 °C or 450 °C), deposition duration (1 min or 3 min), substrate temperature (25 °C or 100 °C), metal type (Zn or Cu), and the volume of solution applied (0.5 mL or 1 mL). Through the examination of ZnPc samples that were deposited for a period of 1 min via sublimation onto a glass substrate held at a temperature of 130 °C, with variations in the temperature of the deposited material from the evaporator, it was observed that at a temperature of 350 °C (Figure 9a), the resulting film exhibited a thin and uniform structure, characterized by a very fine granulation, a minimal height variation of up to ~70 nm, and a low root mean square (RMS) and average roughness of approximately ~4 nm and 3 nm, respectively (Table 3). The grain-size distribution is presented below each AFM image, and the values of the mean values and standard deviations of the minimum diameter (d_min_), maximum diameter (d_max_) and equivalent diameter (d_eqv_) of the grains calculated from the AFM images of the ZnPc and CuPc thin films, depending on the deposition conditions, which are depicted in Table 3.

Adjusting the temperature to 450 °C (Figure 9b) led to a markedly different deposition appearance, characterized by a granular texture, larger grains as compared to previous observations, rounded and well-defined, yet uniformly distributed across the entire examined surface. The surface heights exhibited variations of up to approximately 115 nm, while the RMS and average roughness significantly increased to around 14 nm and 12 nm (Table 3), in contrast to the sample acquired at a sublimation temperature of 350 °C. The rise in the fractal dimension from 2.690 to 2.773 clearly indicates the ZnPc layer deposited transitions to a three-dimensional structure as the sublimation temperature increases.

Increasing the deposition time to 3 min, while keeping the substrate temperature at 130 °C, results in a granular surface characterized by rounded and well-separated grains, observed at the sublimation temperature of 350 °C (Figure 9c). The reduced flow rate in this scenario facilitates adequate time for the three-dimensional growth of crystallites, leading to the formation of larger grains and a more pronounced relief, as evidenced by the elevated fractal dimension value of 2.859. This contrasts with the value of 2.472 recorded for the sample subjected to a sublimation temperature of 450 °C. In a similar manner, it has been noted that with an increase in temperature, the resulting film becomes thicker; however, the elevated flux leads to a greater number of nuclei that fuse more rapidly, effectively filling the valley regions (Figure 9d). This results in a local flattening of the relief within the 10 × 10 µm^2^ scanned area, as evidenced by a decrease in RMS and average roughness from approximately 26 nm and 20 nm (for sample 3 ZnPc) to around 14 nm and 11 nm (for sample 4 ZnPc) (see Table 3).

It was intriguing to examine the outcomes when CuPc was deposited under comparable conditions for a duration of 1 min. At a sublimation temperature of 350 °C, the morphology no longer appears as granular-type, like in the case of ZnPc under the same conditions (Figure 9a), but rather like an acicular network, probably formed of elongated crystallites interspersed with sub-micrometer granules, characterized by sharp edges (Figure 9e). The directional growth of CuPc crystallites, resulting in RMS and average roughness of approximately 23 nm and 17 nm (Table 3), is not present at 450 °C (Figure 9f). At this temperature, the surface relief appears more rounded and smoother, with an RMS and average roughness of around 16 nm and 12 nm (Table 3). However, it becomes significantly more complex due to the presence of small globular formations, leading to a high fractal dimension of 2.735. Despite their differing appearances, both ZnPc and CuPc exhibit a similar tendency to develop a granular relief, characterized by granules that are uniformly distributed across the entire surface examined at 450 °C for 1 min.

The film deposition technique has a significant influence on the characteristics of surface morphology. The 2D AFM topographic images of the films deposited via the drop casting technique, as shown in Figure 10, provide support for this, particularly when compared to the images in Figure 9. Upon the deposition of 0.5 mL of ZnPc solution in formic acid onto a glass substrate at room temperature (Figure 10a), the solvent gradually evaporated, facilitating the aggregation of crystallites with well-defined edges. The morphology observed was characterized by chaos and disorder, featuring substantial islands and profound valleys, with a height difference of 936 nm and an RMS and average roughness of 94 nm and 68 nm, respectively (Table 3). The resulting film in this instance was uneven. When the glass substrate was heated to 100 °C, the solvent evaporation was still relatively slow, but slightly faster than in the previous case, allowing the ZcPc to agglomerate and form large crystalline regions with irregular shapes (Figure 10b). The outcomes yielded a significant height range, reaching up to 890 nm, along with notable RMS and average roughness measuring 130 nm and 101 nm (see Table 3). Nonetheless, in terms of the film’s overall appearance, a notable enhancement in coverage was observed. When the substrate was heated to a temperature of 350 °C, the elevated temperature facilitated solvent evaporation and prevented the excessive growth of individual crystallites (Figure 10c). Thus, the crystallization was more controlled, more nuclei appeared, but with smaller sizes, resulting in a much more uniform and well-compacted layer with a maximum height of ~600 nm, a low RMS and average roughness of around 81 nm and 63 nm (Table 3), and an average isotropy of 56%.

The type of metal utilized is another factor that affects the texture of the surface morphology. When CuPc was substituted for ZcPc, and the film deposition conditions remained unchanged (Figure 10d,e), certain similarities can be noted. At room temperature it was observed the development of rough films, RMS, and average roughness varying from ~94 nm and ~68 nm for 1 ZcPc, RT to ~107 nm and ~85 nm for 7 CuPc, RT) (Table 3). The morphology exhibits agglomeration, characterized by islands of substantial and irregular local thicknesses on the unheated glass substrate (Figure 10d), which can be attributed to the slow evaporation of the solvent and disordered crystallization.

Heating the substrate to 100 °C, where evaporation is faster, leads to preferential nucleation, resulting in more elongated and ordered structures appearing on the surface, some resembling columnar crystallites (Figure 10e). In this instance, as seen in Table 3, the RMS and average roughness are still considerable (~110 nm and ~83 nm), yet the texture exhibits a more directional characteristic compared to the film obtained on the unheated substrate (showing an anisotropy of 40%), the anisotropy in this case being 61%. When comparing the ZnPc film deposited under identical conditions (Figure 10b), it is evident that heating the substrate to 100 °C enhances the uniformity of the deposition for both materials. However, the effect is more subtle for ZnPc (Figure 10b), where the formations remain agglomerated, compact, spheroidal, and lack a distinct orientation. In contrast, for CuPc (Figure 10e), the development of a more ordered and slightly directed columnar morphology was favored.

The surface roughness parameters and grain characteristics presented in Table 3 offer detailed insights into the films’ topographical features, such as average height variations, surface symmetry, and the presence of sharp peaks or deep valleys. In addition to surface morphology, film thickness was determined for ZnPc and CuPc thin films obtained under various deposition conditions using a digital micrometer with a precision of 0.001 mm. Measurements were taken at several points across each film, and the reported values represent the average to assess layer uniformity.

A correlated analysis of morphological and optical parameters reveals a clear relationship between surface roughness and the spectral properties of ZnPc and CuPc thin films. Films prepared by drop casting exhibit the highest roughness values (Sa ≈ 68–101 nm), which is accompanied by a significant decrease in fluorescence intensity (on the order of 10^5^ counts, e.g., 2.5 × 10^5^ for ZnPc at 100 °C). This reduction can be attributed to optical losses induced by surface scattering as well as to possible non-radiative recombination pathways caused by morphological inhomogeneity.

In contrast, films obtained by CSS are characterized by much lower roughness (Sa ≈ 2.7–20 nm), which favors higher fluorescence intensity (up to 7.8 × 10^5^ counts for ZnPc deposited at 450 °C, CSS 3 min). These results confirm that smoother and more compact surfaces promote radiative processes by minimizing optical losses and enhancing light emission. Moreover, a slight shift in the fluorescence bands was observed at higher deposition temperatures (793–797 nm for ZnPc deposited at 350–450 °C, CSS 1 min), suggesting structural rearrangements and improved molecular ordering.

For CuPc, roughness values are generally higher than those of ZnPc, regardless of the deposition method, which correlates with their lower fluorescence intensity (~2–3 × 10^5^ counts). Overall, these findings demonstrate that morphological parameters exert a direct influence on the optical response, and that optimizing the deposition technique—particularly through CSS at elevated temperatures is crucial for achieving thin films with enhanced optoelectronic performance.

## 3. Materials and Methods

### 3.1. Materials

Zinc phthalocyanine (ZnPc) and copper phthalocyanine (CuPc), both with a purity of 96% and purchased from Sigma-Aldrich (Munich, Germany), were used in this study to prepare thin films via two different experimental techniques: (I) drop casting (DC) and (II) close space sublimation (CSS). The purpose of this dual approach was to investigate the influence of deposition conditions on the absorption and fluorescence spectral properties. Formic acid (98% purity) was purchased from Sigma-Aldrich (Munich, Germany) and used as a solvent in experiments. Freshly prepared solutions have been used at moderate concentrations (ranging from 10^−5^ to 10^−6^ M) in appropriate solvents and conducted measurements immediately to minimize aggregation effects and to allow a direct comparison between ZnPc and CuPc.

### 3.2. Drop Casting Deposition Method (DC)

This simple yet effective method involved the controlled deposition of ZnPc and CuPc solutions onto glass substrates with a surface area of 25 mm^2^. The substrates were first subjected to a rigorous chemical cleaning process, being immersed for 24 h in a potassium dichromate (K_2_Cr_2_O_7_) solution, followed by repeated rinsing with distilled water. Subsequently, they were placed in an ultrasonic bath for one hour at a temperature of 50 °C to completely remove residual impurities.

For each material type, individual solutions of ZnPc and CuPc were prepared in formic acid, with an initial concentration of 1 mg/mL (corresponding to a molar concentration of approximately 1.74 mM). This concentration was specifically chosen since it provides the most favorable optical and morphological properties, as already investigated in detail by V. Furtună (PhD thesis “Development of Photovoltaic Devices Based on Organic/Inorganic Semiconductors”, 2022) [45]. The relevant information from the thesis confirms that higher solute concentrations tend to promote molecular aggregation, leading to spectral broadening and red-shifted absorption and emission features, consistent with our present findings. From each solution, 0.5 mL was dropped onto the prepared substrates. The resulting samples were dried under two different thermal conditions to observe the influence of temperature on the film formation process: one at room temperature-RT (≈25 °C), tagged as sample 1 for ZnPc and sample 7 for CuPc, and the other at 100 °C for one hour in atmosphere (samples 2 and 8, respectively).

### 3.3. Close Space Sublimation Method (CSS)

The second method, significantly more sophisticated and suitable for obtaining films with better homogeneity and density, was the CSS technique. In this case, the substrate temperature was kept constant at 130 °C for both types of materials (ZnPc and CuPc). To optimize the technological deposition conditions, two sets of experiments were conducted by varying the evaporator temperature (350 and 450 °C), the evaporation time (1 and 3 min) and high vacuum 10^−5^ Bar. According to the literature [46], both ZnPc and CuPc exhibit thermal stability up to ~500–600 °C before significant degradation occurs. Therefore, the maximum evaporator temperature used in our CSS experiments (450 °C), combined with the short deposition time (on the order of a few minutes), is well below the threshold for structural decomposition, ensuring that the molecular integrity of the phthalocyanines is preserved during the sublimation process.

The first set included two samples obtained at different evaporation temperatures: 350 °C (sample 15) and 450 °C (sample 16), with a constant evaporation time of 3 min. The second set followed the same temperature regime (350 °C–sample 17 and 450 °C–sample 18), but with a reduced evaporation time of 1 min. In all cases, the substrate temperature remained unchanged, allowing for the evaluation of the influence of evaporation parameters on the quality of the resulting films.

### 3.4. Methods

The electronic absorption spectra were collected on a SPECORD 210 Plus spectrometer (Analytik Jena, Jena, Germany), using quartz cells with the path length of 10 mm. The double-beam configuration enables simultaneous measurement of the sample and the reference, reducing the effects of light source and detector fluctuations. Baseline correction is achieved by using a blank in the reference cuvette and through automatic software compensation, ensuring excellent stability and minimal noise. The UV-Vis absorption spectra were recorded in the 300–950 nm range. Although, the instrument allows measurements between 200 and 1100 nm, the UV region below 300 nm was omitted due to strong absorption of the glass substrates used for thin-film deposition. Fluorescence spectra were collected up to 850 nm, corresponding to the upper sensitivity limit of the detector.

Steady-state fluorescence spectra were recorded on an Edinburgh FS5 spectrometer (Edinburgh Instruments, Livingston, UK) using a xenon lamp as the excitation source. All fluorescence measurements were conducted under strictly identical experimental conditions (e.g., same excitation wavelength, slit widths, solvent (FA), concentration (29 µM), and cuvette pathlength), allowing for a reliable relative comparison between samples. Emission scans were performed in the 350–850 nm range with a step size of 1 nm. The excitation and emission slits were set to approximately 5 nm and 10 nm, respectively, and signal correction was carried out using the reference detector file.

Time-resolved photoluminescence measurements were performed on an FLS 980 spectrometer (Edinburgh Instruments, Livingston, UK) using the time-correlated single-photon counting (TCSPC) technique with a nanosecond diode laser excitation source at 375 nm. The detection range of the photomultiplier tube (PMT) extended up to 850 nm, ensuring full coverage of the near-infrared emission band. All measurements were performed at room temperature.

Measurements utilizing atomic force microscopy were performed with an NTEGRA system provided by NT-MDT Spectrum Instruments Company (Zelenograd, Moscow, Russia). An NSG 10 cantilever (TipsNano OÜ, Tallinn, Estonia) featuring a resonance frequency of 190 kHz and a normal spring constant of 14.1 N/m was utilized to analyze the surface texture of the samples under ambient conditions at 23 °C. The operational methodology employed was in tapping mode. The program Nova 1.1.1.19891 (NT-MDT Spectrum Instruments in Zelenograd, Moscow, Russia) was utilized for capturing images on surfaces ranging from 60 × 60 µm^2^ to 5 × 5 µm^2^. A scanning area measuring 10 × 10 µm^2^ was designated as representative. The surface analysis was performed using ImageAnalysis software (version 3.5.0.19892) and MountainsSPIP^®^ software (version 10, Academic license) for scanning probe microscopy were utilized to conduct a comprehensive analysis of the surfaces.

## 4. Conclusions

This study described zinc phthalocyanine (ZnPc) and copper phthalocyanine (CuPc) thin films obtained using two different experimental methods: the drop casting method (DC), and (II) the close space sublimation (CSS) method. Experiments have shown that the best results are obtained with the CSS method because the resulting ZnPc and CuPc thin films exhibit improved surface uniformity and molecular ordering compared to those obtained via the drop casting method. The controlled environment in CSS allows for more precise deposition, resulting in thinner, more homogeneous films with fewer defects. Additionally, CSS enables better adherence to the substrate and promotes stronger intermolecular interactions, which are critical for enhancing the optical and electronic properties of the phthalocyanine films. In addition, the experimental results demonstrate that the films exhibit a high optical transparency of approximately 90%. For both CuPc and ZnPc, the Q-band is well defined and located around 680 nm, while the fluorescence maximum appears at ~825 nm with the highest emission intensity. In contrast, films obtained by the drop casting method display a Q-band at nearly the same position (~675 nm) and a fluorescence maximum also around 825 nm; however, their transparency does not exceed 70%. These qualities make the CSS-derived films more suitable for applications in optoelectronic devices, such as organic photovoltaics and sensors. An important aspect to highlight is the crucial role of film thickness in determining the fluorescence maximum (CSS method). For films with a thickness of approximately 100 nm, the emission peak is observed around 795 nm, whereas for thicker films exceeding 300 nm, the fluorescence maximum shifts toward ~825 nm. This thickness-dependent red shift can be attributed to enhanced molecular aggregation and stronger intermolecular interactions in thicker films, which significantly influence the optical response of phthalocyanine layers. The AFM study results highlighted the major influence of the deposition method. The films obtained by sublimation deposition exhibited lower roughness and adjustable morphology induced by the process parameters. In contrast, films obtained by drop casting exhibited increased roughness, a more rugged topography, and less controllable properties. The findings indicate that the microstructure and properties of Zn and Cu phthalocyanine films are influenced by the interplay between the deposition rate and molecular mobility on the substrate surface, which can be adjusted through variations in temperature and time. It is important to note that this study was limited by the available experimental setup. Therefore, only relative photoluminescence (PL) intensities were considered. Absolute quantum yield and time-resolved decay measurements are planned for future investigations to provide a more complete evaluation of the photophysical properties.

## Figures and Tables

**Table 1:** Comparison of physical properties of ZnPc and CuPc.

Property	ZnPc (Zinc Phthalocyanine)	CuPc (Copper Phthalocyanine)
Molecular Formula	C_32_H_16_N_8_Zn	C_32_H_16_N_8_Cu
Central Metal	Zn^2+^	Cu^2+^
Molecular Weight (g/mol)	~577.92	~576.1
Molecular Geometry	Planar and rigid [8]	Approximately planar, slightly distorted [9]
Thermal Stability	Up to ~600 °C [10]	Up to ~500 °C [11]
Spectral Shift (Q-band)	Red-shifted [12]	Blue-shifted [13]
Electronic Configuration	Closed-shell d10 for Zn [14]	Open-shell d9 for Cu [15]
Absorption Peak (Q-band)	Sharp, centered ~670–680 nm	Broader, centered ~620–640 nm
Fluorescence Quantum Yield	Relatively high (up to ~10%)	Very low fluorescence [9]
Chemical Stability	Sensitive to some solvents and conditions	High stability against oxidation and humidity
Charge Transport	Efficient electron transport due to crystalline films	Moderate transport, but better environmental stability
Band Gap (eV)	~1.7–1.9 [16,17]	~1.5–1.7 [18]

**Table 2:** Summarizing the key absorption and fluorescence parameters for ZnPc and CuPc thin films obtained by different deposition methods.

Sample	Material	Deposition Method	Temp (°C)	Absorption Band (Q-Band, nm)	Fluorescence Band (nm)	Fluorescence Intensity (Counts)
1	ZnPc	Drop casting	RT	~674	825	~5.2 × 10^5^
2	ZnPc	Drop casting	100	~674	825	~2.5 × 10^5^
3	ZnPc	CSS (3 min)	350	~675	826	~4.8 × 10^5^
4	ZnPc	CSS (3 min)	450	-	824	~7.8 × 10^5^
15	ZnPc	CSS (1 min)	450	~676	797	~1.2 × 10^5^
16	ZnPc	CSS (1 min)	350	~675	793	~1.0 × 10^5^
7	CuPc	Drop casting	RT	~688	822	~2.3 × 10^5^
8	CuPc	Drop casting	100	~688	822	~2.1 × 10^5^
17	CuPc	CSS (1 min)	450	~689	823	~7.2 × 10^5^
18	CuPc	CSS (1 min)	350	~689	822	~3.2 × 10^5^

**Table 3:** Summarizing the film thickness and surface roughness parameters (Sq—root mean square (RMS) roughness, Sa—average roughness), mean values and standard deviations of the minimum diameter (d_min_), maximum diameter (d_max_) and equivalent diameter (d_eqv_) of the grains calculated from the AFM images of the ZnPc and CuPc thin films, depending on the deposition conditions.

Sample	Material	Deposition Method	Temp (°C)	Thickness (µm)	Sq (nm)	Sa (nm)	d_min_ (nm)	d_max_ (nm)	d_eqv_ (nm)
16	ZnPc	CSS (1 min)	350	2	4.24	2.77	210 ± 77	361 ± 132	276 ± 97
15	ZnPc	CSS (1 min)	450	11	14.62	11.71	382 ± 152	642 ± 196	494 ± 165
3	ZnPc	CSS (3 min)	350	6	26.49	20.27	331 ± 126	668 ± 281	482 ± 180
4	ZnPc	CSS (3 min)	450	-	14.27	11.00	327 ± 112	612 ± 175	459 ± 123
18	CuPc	CSS (1 min)	350	4	22.83	17.33	234 ± 60	436 ± 108	326 ± 70
17	CuPc	CSS (1 min)	450	6	15.86	12.31	160 ± 56	283 ± 98	214 ± 69
1	ZnPc	Drop casting	RT	2	94.02	68.26	203 ± 46	368 ± 72	278 ± 44
2	ZnPc	Drop casting	100	8	130.60	101.00	181 ± 69	322 ± 121	243 ± 86
13	ZnPc	Drop casting	350	-	81.39	63.34	279 ± 110	565 ± 229	407 ± 149
7	CuPc	Drop casting	RT	5	107.20	84.87	335 ± 86	696 ± 182	496 ± 95
8	CuPc	Drop casting	100	15	110.30	82.84	220 ± 56	405 ± 101	304 ± 64

## Data Availability

Data will be made available on request.
